# 2D Gallium Sulfide-Based 1D Photonic Crystal Biosensor for Glucose Concentration Detection

**DOI:** 10.3390/ma16134621

**Published:** 2023-06-27

**Authors:** Abdelkader Abderrahmane, Khaled Senouci, Belkacem Hachemi, Pil Ju Ko

**Affiliations:** 1 Department of Electrical Engineering, Chosun University, 375, Seosuk-dong, Dong-gu, Gwangju 501-759, Republic of Korea; abderrahmane.abdelkader@gmail.com; 2 Laboratoire de Structure, Elaboration et Application des Matériaux Moléculaires (SEA2M), Université Abdelhamid Ibn Badis Mostaganem, B.P. 227, Mostaganem 27000, Algeria; khaled.senouci@univ-mosta.dz; 3 Division Architecture et Système Multimédia, Centre de Développement des Technologies Avancées, Alger 16000, Algeria; bhachemi@cdta.dz

**Keywords:** 1D photonic crystal, Bragg reflector, diabetes, biosensor, glucose detector

## Abstract

Unidimensional photonic crystal-based biosensors have gained much attention in the area of blood glucose measurement. In this paper, we propose two novel designs based on two-dimensional (2D) Van der Waals materials. The first 1D photonic crystal design consists of multilayers of 2D gallium sulfide and 2D muscovite mica [GaS/Mica]*^N^*D[GaS/Mica]*^N^*, and the second design consists of multilayers of 2D gallium sulfide [GaS/G]*^N^*D[GaS/G]*^N^*. We conducted a numerical analysis using the transfer matrix method to investigate the properties of photonic crystals, both with and without defect layers, in order to assess their suitability for biosensing applications. The biosensors’ performances were investigated as a function of glucose concentration, revealing a high sensitivity of 832 nm/RIU, a notable figure-of-merit of 1.46 × 10^5^ RIU^−1^, a Q-factor exceeding 10^5^, and a minimum limit of detection of 3.4 × 10^−7^ RIU. Finally, we modified the [GaS/G]*^N^*D[GaS/G]*^N^*structure in order to enhance the sensitivity nearly 5-fold. The proposed biosensors offer the advantage of being label-free, making them promising platforms for the sensitive and reliable detection of blood glucose levels.

## 1. Introduction

In recent years, diabetes has become a major global health concern that affects a large number of people around the globe. According to statistics, 642 million individuals may be affected by diabetes by the year 2040 [1]. This disease arises when the body is unable to produce insulin, which is essential for glucose conversion into energy, known as type 1 diabetes, or when the body either does not produce enough insulin or is unable to use it properly, known as type 2 diabetes [2]. Diabetes can cause many complications, including cardiovascular disease, vision problems, kidney failure, brain dysfunction, and even limb amputations [3]. Although there is no cure for diabetes, patients have to manage their condition effectively through various measures and should also monitor their blood sugar levels regularly. Therefore, it is crucial to develop sensitive, reliable, cost-effective, and timely diabetes detection biosensors, as early detection can lead to earlier treatment and a higher chance of avoiding the above-mentioned complications related to the disease.

Photonic crystals (PCs) have been explored as a potential solution for the development of biosensors with the aim of measuring blood glucose concentrations with high precision and high sensitivity [4,5,6,7]. PCs consist of a periodic structure with alternating refractive indices, which leads to the formation of photonic band gaps and the ability to control electromagnetic wave propagation, i.e., transmit or reflect certain wavelengths [8]. Specific optical properties of the PCs can be tuned by tailoring their geometries, allowing the development of photonic devices for a variety of applications, including temperature sensing [9], biosensing [10], chemical and biochemical sensing [11], pressure detection [12], and gas sensing [13]. Unidimensional (1D) photonic crystals, in particular, have been extensively studied for biosensing applications due to their simple structures and high sensitivities [14]. The biosensors based on 1D PCs consist of a series of alternating layers of materials and one or multiple target layers. The variation of the refractive index of the defect layer results in a change in the reflectance or transmittance of the crystal. By measuring this change, the biosensors can detect specific biological molecules or analytes. Several biosensing applications have been reported using 1D PCs, such as the detection of extracellular vesicles [15], viruses [16], bacteria [17], proteins, DNA, and RNA [10]. One-dimensional photonic crystal-based biosensors for glucose monitoring have shown promising results in terms of sensitivity and Q-factor [18].

Two-dimensional (2D) materials, consisting of atomically thin layers, are the best candidates for the fabrication of 1D photonic crystals thanks to their unique and tunable optical properties, as previously reported in our research [19,20,21]. Furthermore, they can be easily deposited and controlled in terms of thickness and number of layers, allowing for precise tuning of the photonic bandgap. Two-dimensional muscovite mica (KAl_2_(Si_3_AlO_10_)(OH)_2_) and 2D gallium sulfide (GaS) have gained significant attention in electronic device applications [22,23]; however, to the best of our knowledge, no reports have yet been published on 1D photonic crystal based on muscovite mica and GaS. GaS exhibits a wide direct band gap of ~3.1 eV in its monolayer form and ~2.6 eV in its bulk form [24], a high refractive index of 2.7152 at a wavelength of 633 nm [25], and a very broad spectral range, making it an excellent candidate for 1D photonic crystals. On the other hand, 2D muscovite mica is a transparent material with a wide direct band gap of 5.09 eV [26]. It has a slightly low refractive index (n_α_ = 1.552 − 1.576 n_β_ = 1.582 − 1.615 n_γ_ = 1.587 − 1.618), as reported by H. Ghafari et al. [27]. In addition, muscovite mica has high thermal and chemical stability [28].

In this paper, we report for the first time two novel designs of 1D photonic crystal biosensors based on multilayered 2D materials, specifically the [GaS/Mica]*^N^*, [GaS/Mica]*^N^*D[GaS/Mica]*^N^*, and [GaS/G]*^N^*D[GaS/G]*^N^* structures, where G refers to target layers. The sensing mechanism is based on the detection of changes in the optical properties of the PCs due to variations in the glucose solution concentration. We investigated numerically the photonic properties of the 1D PCs with respect to the periodic number N, target concentrations, defect thickness, and incidence angle of light. The introduction of prism and gold layers to the [GaS/G]*^N^*D[GaS/G]*^N^*-based biosensor improved the sensitivity and other parameters. The successful integration of 2D materials within 1D photonic crystal structures highlights a promising avenue of research in biosensing.

## 2. Biosensor Designs and Theoretical Formulation

**Figure 1:**
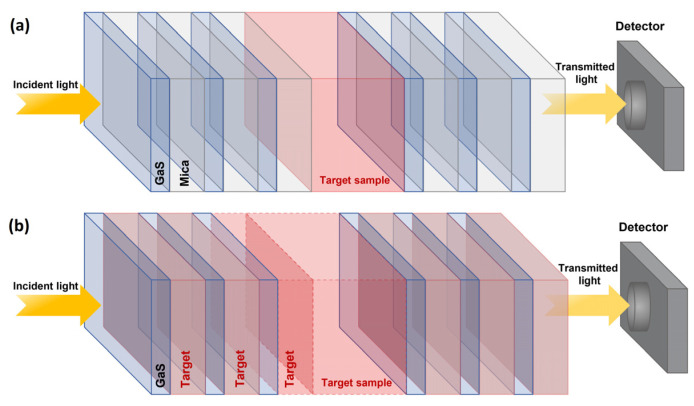
Schematic of the proposed 1D photonic crystal-based biosensors, (**a**) [GaS/Mica]^N^D[GaS/Mica]^N^ structure and (**b**) [GaS/G]^N^D[GaS/G]^N^ structure.

Figure 1 illustrates the proposed designs of the biosensors. The design depicted in Figure 1a (biosensor 1) consists of a periodic arrangement of [GaS/Mica] layers with a defect layer positioned in the center of the structure and in which glucose solution can be injected. The design depicted in Figure 1b (biosensor 2) is composed of a periodic arrangement of [GaS/G] layers with a defect layer positioned in the center of the structure. Here, the symbol G refers to cavity layers in which solution can be filled, i.e., the biosensor can be immersed in the glucose solution, forming the target layers shown in Figure 1b. The thicknesses of GaS and mica were calculated, respectively, as 73.9 and 129 nm at a wavelength ($\lambda_{0}$) of 800 nm, using the equation $d=\lambda_{0}/4n$, where n represents the refractive index. The refractive index of GaS can be deduced from the dispersion formula given by [28]:

(1) $$n_{GaS}=7.12996+\frac{0.26073}{\lambda^{2}-0.04627}+\frac{127.335}{\lambda^{2}-258.431}$$

The refractive index of the mica flake ($n_{Mica}$) was chosen as $n_{Mica}=1.55$, according to reference [29]. Using the same approximation, the thickness of the G layer (Figure 1b) was calculated as 150.1 nm, assuming a glucose concentration of zero. The relationship between the glucose concentration (C [g/L]) and the average refractive index ($n_{sugar}$) is given by a linear function of the form [30]:

(2) $$n_{sugar}=0.00011889\times C+1.33230545$$

The interaction between light and structure 1, as well as structure 2, can be simulated using the transfer matrix method, as provided by [31]:

(3) $$M=\overset{2N+1}{\underset{j=1}{\prod }}M_{j}={\left(M_{GaS}M_{Mica}\right)}^{N}M_{D}{\left(M_{GaS}M_{Mica}\right)}^{2N+1}=\left(\begin{array}{cc} m_{11} & m_{12}\\ m_{21} & m_{22} \end{array}\right)$$

(4) $$M=\overset{2N+1}{\underset{j=1}{\prod }}M_{j}={\left(M_{GaS}M_{G}\right)}^{N}M_{D}{\left(M_{GaS}M_{G}\right)}^{2N+1}=\left(\begin{array}{cc} m_{11} & m_{12}\\ m_{21} & m_{22} \end{array}\right)$$

where *N* refers to the periodic number. The parameters $m_{11}$, $m_{12}$, $m_{21}$, and $m_{22}$ are the elements of the total transfer matrix. $M_{j}$ is the characteristic matrix of each layer and is given by:

(5) $$M_{j}=\left(\begin{array}{cc} cos\left(\delta_{j}\right) & \frac{-i\;sin\left(\delta_{j}\right)}{\gamma_{j}}\\ -i\;\gamma_{j}\;sin\left(\delta_{j}\right) & cos\left(\delta_{j}\right) \end{array}\right)$$

where $\delta_{j}=\left(\omega /c\right)\;n_{j}\;d_{j}\;cos\left(\theta_{j}\right)$, $n_{j}$ and $d_{j}$ are, respectively, the refractive index and the thickness of the *j*th layer, $\theta_{j}$ is the incidence angle at the *j*th layer, ω is the angular velocity, and c is the speed of light. The parameter $\gamma_{j}$ is given by the equation $\gamma_{j}=n_{j}\times \cos\left(\theta_{j}\right)$ in the case of the transverse electric mode (TE), and by $\gamma_{j}=n_{j}\;/\cos\left(\theta_{j}\right)$ in the case of the transverse magnetic mode (TM).

The reflection and transmittance coefficients are defined by:

(6) $$r=\frac{\gamma_{1}\left(m_{11}+\gamma_{t}\;m_{12}\right)-\left(m_{21}+\gamma_{t}\;m_{22}\right)\;}{\gamma_{1}\left(m_{11}+\gamma_{t}\;m_{12}\right)+\left(m_{21}+\gamma_{t}\;m_{22}\right)}$$

(7) $$t=\;\frac{2\gamma_{1}\;}{\gamma_{1}\left(m_{11}+\gamma_{t}\;m_{12}\right)+\left(m_{21}+\gamma_{t}\;m_{22}\right)}$$

where $\gamma_{1}$ and $\gamma_{t}$ correspond to $\gamma_{j}$ at the first (*j* = 1) and the last layer (*j* = 2N + 1), respectively. Finally, the reflection (R) and the transmittance (T) are given by $R={\left|r\right|}^{2}$ and $T=\left(\gamma_{t}/\gamma_{1}\right){\left|t\right|}^{2}$, respectively.

## 3. Numerical Results and Discussion

### 3.1. Optical Characteristics of the 1D PCs with and without a Defect

**Figure 2:**
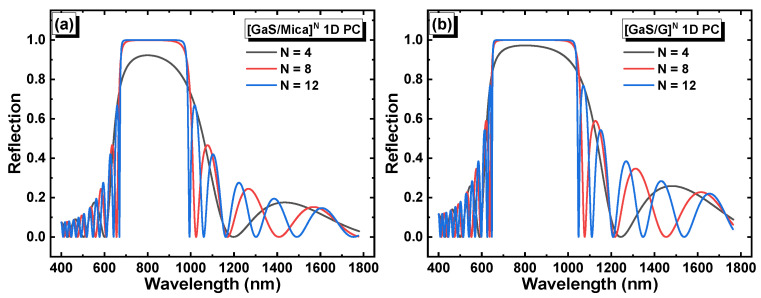
Reflection versus the wavelength and period number in (**a**) [GaS/Mica]*^N^* and (**b**) [GaS/G]*^N^*.

In the absence of a defect layer, the 1D PCs are expected to exhibit a photonic band gap, which corresponds to the range of wavelengths at which light transmission is prohibited or strongly suppressed. Figure 2a,b depict the reflection of light as a function of the wavelength and the number of periods *N* in the structures [GaS/Mica]*^N^* and [GaS/G]*^N^*, respectively. In the second structure, layer G refers to a solution with zero glucose concentration (i.e., distilled water in this case). 

**Figure 3:**
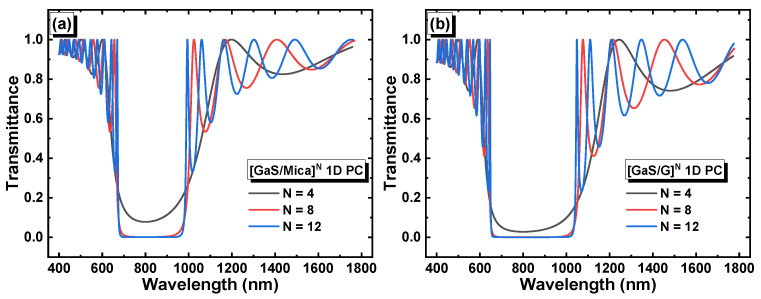
Transmittance versus the wavelength and period number in (**a**) [GaS/Mica]*^N^* and (**b**) [GaS/G]*^N^*.

The corresponding transmittance in each structure is shown in Figure 3a,b. The [GaS/Mica]*^N^* and [GaS/G]*^N^* structures had band gap widths of 0.61 eV and 0.73 eV, respectively, at a period number of 12. Additionally, both structures, [GaS/Mica]^12^ and [GaS/G]^12^, exhibited well-defined band gaps in the visible to near infrared range, which makes them suitable for opto-sensing applications. The significant values of the photonic band gaps were due to the substantial refractive index difference between GaS and mica in the [GaS/Mica]^12^ structure, as well as the refractive index difference between GaS and distilled water in the [GaS/Water]^12^ structure.

**Figure 4:**
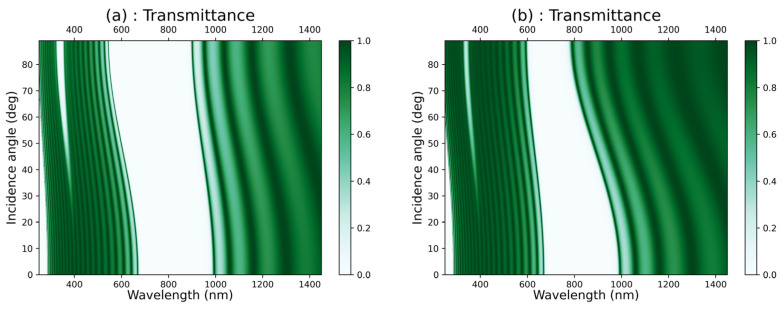
Colormap plot of the transmittance versus the wavelength and the incidence angle of light in [GaS/Mica]*^12^* in the case of (**a**) TE polarization mode and (**b**) TM polarization mode.

**Figure 5:**
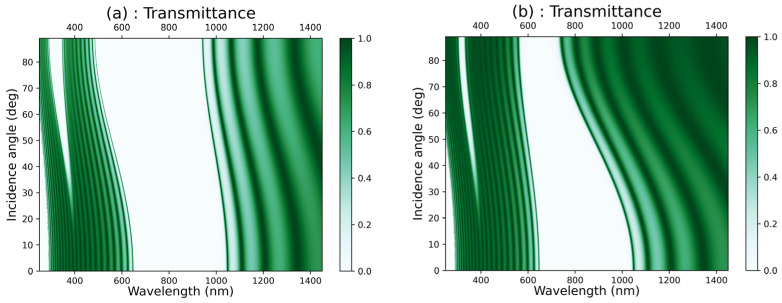
Colormap plot of the transmittance versus the wavelength and the incidence angle of light in [GaS/water]*^12^* in the case of (**a**) TE polarization mode and (**b**) TM polarization mode.

We examined the impact of the incidence angle of light on the transmittance characteristics in the [GaS/Mica]^12^ structure in the case of the TE polarization mode (Figure 4a) and the TM polarization mode (Figure 4b). Similarly, we investigated the effect of the incidence angle on the transmittance in the [GaS/Water]^12^ structure in the case of the TE polarization mode (Figure 5a) and the TM polarization mode (Figure 5b). We observed that in the case of TE polarization, the photonic band gaps in both structures widen and shift towards shorter wavelengths when the incidence angle increases from zero to 89°. Contrarily, in the case of TM polarization, the photonic band gaps narrow as the incidence angle increases in both structures.

**Figure 6:**
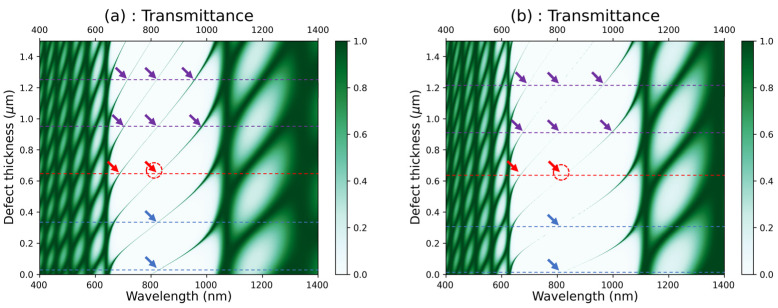
Colormap plot of the transmittance versus the wavelength and the defect thickness in (**a**) [GaS/Mica]^6^D[GaS/Mica]^6^ and (**b**) [GaS/water]^6^D[GaS/water]^6^; D refers to distilled water.

When a defect layer is created inside the PCs, the transmittance exhibits different behavior compared to the case without a defect. In fact, the presence of the defect introduces additional optical modes and modifies the optical characteristics within the structure. As a result, peaks and dips appear in the transmittance characteristic at specific wavelengths corresponding to the resonant modes. The resonant modes in PCs depend on various factors, such as the PC structure, the wavelength, and the angle of the incident light on the crystal. Figure 6a,b display the colormap plots of the transmittance in [GaS/Mica]^6^D[GaS/Mica]^6^ and [GaS/water]^6^D[GaS/water]^6^, respectively, with respect to the wavelength and defect thickness. Here, D refers to a zero concentration of glucose, which corresponds to distilled water. In the colormap plots, single resonance modes occur at positions denoted by blue arrows, double resonance modes at positions denoted by red arrows, and triple resonance modes at positions denoted by purple arrows. Assuming the resonance wavelength lies in the middle of the photonic band gap (around 800 nm), the [GaS/Mica]^6^D[GaS/Mica]^6^ structure exhibits single resonance modes at defect layer thicknesses of 25 nm and 320 nm. It shows a double resonance mode at a defect layer thickness of 630 nm and triple resonance modes at defect layer thicknesses of 940 nm and 1245 nm. On the other hand, the [GaS/water]^6^D[GaS/water]^6^ structure exhibits single resonance modes at defect layer thicknesses of 15 nm and 310 nm. It shows a double resonance mode at a defect layer thickness of 640 nm. Finally, triple resonance modes occur at defect layer thicknesses of 915 nm and 1220 nm. In the subsequent calculations, we have kept the defect thickness fixed at 640 nm for both studied structures, at positions denoted by red dashed circles in Figure 6a,b.

**Figure 7:**
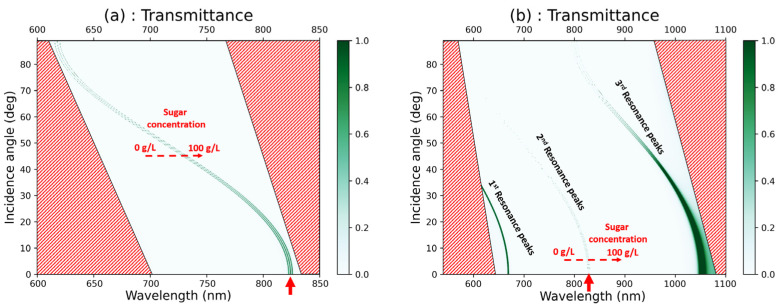
Superimposition of colormap plots of the transmittance, calculated at different values of glucose concentration, versus the wavelength and the incidence angle of light in (**a**) [GaS/Mica]^6^D[GaS/Mica]^6^ and (**b**) [GaS/G]^6^D[GaS/G]^6^.

The optical characteristics of [GaS/Mica]^6^D[GaS/Mica]^6^ and [GaS/G]^6^D[GaS/G]^6^ were studied at different values of glucose concentration (0, 50 g/L, and 100 g/L). It is important to note that both D and G layers can be filled with the target solution, but they have thicknesses of 640 nm and 150.1 nm, respectively. The values of the glucose concentration were chosen to be large enough to illustrate the shift of the resonance peaks. Figure 7a,b represent the superimposition of colormap plots, illustrating the transmittance at various glucose concentration levels as a function of wavelength and incidence angle of light in [GaS/Mica]^6^D[GaS/Mica]^6^ and [GaS/G]^6^D[GaS/G]^6^, respectively. Areas highlighted with red lines in Figure 7a,b indicate non-relevant regions. As can be seen in Figure 7a, it is easy to differentiate between the resonance peaks associated with varying glucose concentrations. In other words, the shift of the resonance peak is clearly discernible. In Figure 7b, three groups of peaks can be observed, namely the first resonance peaks, the second resonance peaks, and the third resonance peaks, which are associated with varying glucose concentrations. Among these, only the peak in the middle exhibits a clear shift due to its very narrow width. Therefore, in the subsequent section, we will focus on the peaks observed at around 820 nm (indicated by the red arrows in the same figures) and analyze their shift as a function of the incidence angle of light and solution concentration. As the incidence angle of light increases, the peaks of the resonance modes shift to lower wavelengths. The shift can be explained by the Bragg–Snell equation [32] given by $m\lambda =2\pi \sqrt{n_{eff}^{2}-sin^{2}\theta }$, where m is the constructive diffraction order, d is the period, $n_{eff}$ is the effective refractive index, and θ is the incidence angle of light. The peaks narrow as the incidence angle increases, indicating a low full width half maximum (FWHM) and thus better biosensing performance.

### 3.2. Biosensing Performance of 1D PCs

**Figure 8:**
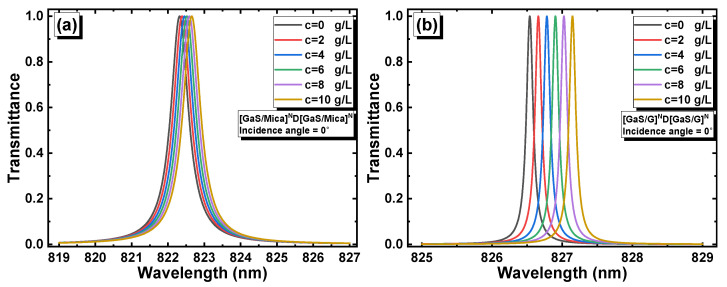

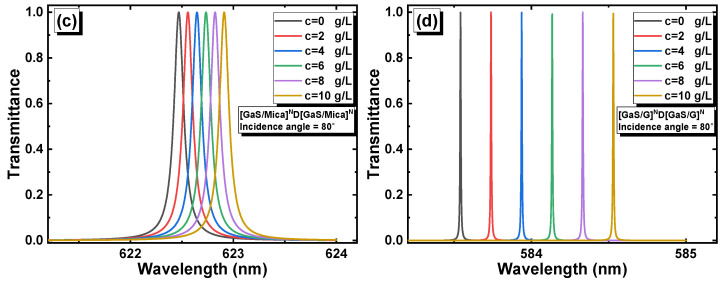
Resonance peak shifts as a function of the glucose concentration in (**a**) [GaS/Mica]^6^D[GaS/Mica]^6^ at a normal incidence angle; (**b**) [GaS/G]^6^D[GaS/G]^6^ at a normal incidence angle; (**c**) [GaS/Mica]^6^D[GaS/Mica]^6^ at an incidence angle of 80°; and (**d**) [GaS/G]^6^D[GaS/G]^6^ at an incidence angle of 80°.

Diabetes was defined in accordance with the American Diabetes Association criteria [33], which specify that fasting blood plasma glucose levels are ≥126 mg/dL (≥1.26 g/L) and non-fasting plasma glucose levels are ≥200 mg/dL (≥2 g/L). In this study, we established a fixed threshold value for the glucose concentration at C = 2 g/L. The performance of the 1D PC biosensors was investigated at this threshold value as well as at higher concentrations (C = 0, 2 g/L, 4 g/L, 6 g/L, 8 g/L, and 10 g/L). We examined the shift of the resonance peaks in [GaS/Mica]^6^D[GaS/Mica]^6^ and [GaS/G]^6^D[GaS/G]^6^ as a function of the glucose concentration at incidence angles of 0° and 80° (Figure 8a–d). The resonance peaks were red-shifted as the concentration increased according to the standing wave condition [34] given by $\Delta =m\lambda =n_{eff}G$, where Δ is the optical path difference, m is an integer, $n_{eff}$ is the effective refractive index which changes linearly with the glucose concentration, and G represents the geometrical path difference. The resonance peaks in the [GaS/G]^6^D[GaS/G]^6^ structure were narrower compared to those in the [GaS/Mica]^6^D[GaS/Mica]^6^ structure, which corresponded to low FWHMs. As the incidence angle increased to 80°, the FWHMs of the resonance peaks in both structures decreased. This indicated that the peaks became narrower, suggesting improved resolution and potentially enhanced biosensing performance.

**Figure 9:**
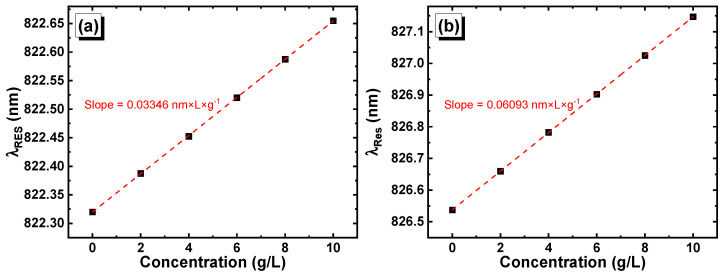
Position of the resonance peak versus the glucose concentration in (**a**) [GaS/Mica]^6^D[GaS/Mica]^6^ and (**b**) [GaS/G]^6^D[GaS/G]^6^.

The shift of the resonance peaks ($\lambda_{RES}$) in [GaS/Mica]^6^D[GaS/Mica]^6^ and [GaS/G]^6^D[GaS/G]^6^ as a function of the glucose concentration at a normal incidence angle of light changed linearly with the variation of the glucose concentration, as is shown in Figure 9a,b, respectively. Fitting plots of $\lambda_{RES}\left(C\right)$, where $\lambda_{RES}$ represents the resonance wavelength and C represents the glucose concentration, leads to the following mathematical equations: $\lambda_{RES}\left(C\right)=0.03346\times C+822.32$ and $\lambda_{RES}\left(C\right)=0.06093\times C+826.54$ in [GaS/Mica]^6^D[GaS/Mica]^6^ and [GaS/G]^6^D[GaS/G]^6^, respectively.

**Figure 10:**
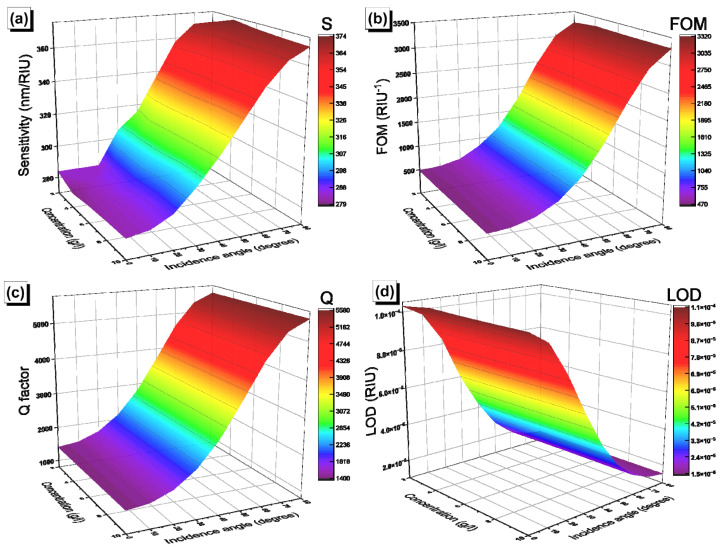
Glucose concentration and incidence angle of light dependence of (**a**) the sensitivity; (**b**) FOM; (**c**) Q-factor; and (**d**) LOD in [GaS/Mica]^6^D[GaS/Mica]^6^.

**Figure 11:**
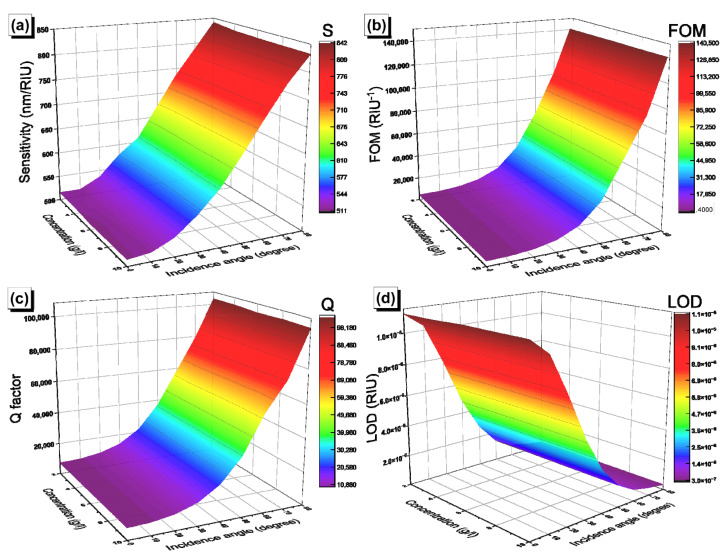
Glucose concentration and incidence angle of light dependence of (**a**) the sensitivity; (**b**) FOM; (**c**) Q-factor; and (**d**) LOD in [GaS/G]^6^D[GaS/G]^6^.

The performance of our biosensors was evaluated by calculating the sensitivity (S), the figure of merit (FOM), the quality factor (Q), and the limit of detection (LOD). The sensitivity was calculated using the equation $S=\Delta \lambda_{RES}/\Delta n_{c}=\left(\lambda_{RES}^{C=x}-\lambda_{RES}^{C=0}\right)/\left(n_{C=x}-n_{C=0}\right)$, where x represents the value of the concentration, which varies between 2 to 10 g/L; $\lambda_{RES}^{C=x}$ is the position of the resonance peak at concentration x; and $n_{C}$ is the corresponding refractive index of the solution. The variation of the sensitivity as a function of the glucose concentration and incidence angle in [GaS/Mica]^6^D[GaS/Mica]^6^ and [GaS/G]^6^D[GaS/G]^6^ is shown, respectively, in Figure 10a and Figure 11a. The sensitivity of each biosensor shows a linear dependence on the concentration and a sigmoid-like dependence on the incidence angle, with maximum values of around 370 nm/RIU and 832 nm/RIU in [GaS/Mica]^6^D[GaS/Mica]^6^ and [GaS/G]^6^D[GaS/G]^6^, respectively. In addition to the sensitivity, the figure of merit (FOM), the quality factor (Q), and the limit of detection (LOD) are given by the equations:

(8) $$FOM=\frac{S}{\lambda_{FWHM}}$$

(9) $$Q=\frac{\lambda_{RES}}{\lambda_{FWHM}}$$

(10) $$LOD=\frac{\lambda_{c}}{20\times S\times Q}$$

where $\lambda_{FWHM}$ is the full width half maximum (FWHM) of the defect mode peak. The higher the values of FOM and Q and the lower the LOD value, the better the performance of the biosensors. Figure 10b and Figure 11b show the variation of the FOM as a function of the glucose concentration. The FOM increases to values of 3306 RIU^−1^ and 1.46 × 10^5^ RIU^−1^ in [GaS/Mica]^6^D[GaS/Mica]^6^ and [GaS/G]^6^D[GaS/G]^6^, respectively, as the incidence angle of light increases to 80°. Similarly, the Q-factor rises as a function of the incidence angle in [GaS/Mica]^6^D[GaS/Mica]^6^ (Figure 10c) and [GaS/G]^6^D[GaS/G]^6^ (Figure 11c), with a maximum value that exceeds 10^5^ in the [GaS/G]^6^D[GaS/G]^6^ structure. Finally, the LOD represented in Figure 10d and Figure 11d is independent of the glucose concentration and decreases with increasing incidence angle of light, with minimum values of 1.5 × 10^−5^ and 3.4 × 10^−7^. The [GaS/G]^6^D[GaS/G]^6^ structure was designed so as to have more sensitivity to minute changes in the glucose concentration. In fact, both the defect layer and the G layer experience variations in the refractive index upon injection of a glucose solution.

### 3.3. Biosensing Performance in the Near-Infrared Range

**Figure 12:**
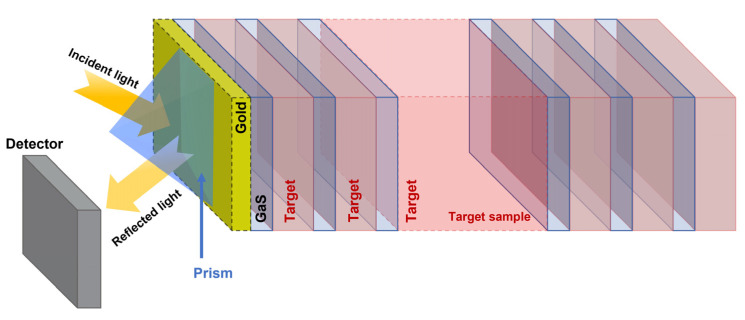
Schematic of prism/Au/1D photonic crystal-based biosensors.

Finally, we investigated the biosensing performance of the [GaS/G]^6^D[GaS/G]^6^ structure in the near-infrared range by adding prism (BK7 glass) and 20 nm thick gold (Au) layer. The proposed design is represented schematically in Figure 12. The refractive index of the BK7 glass prism ($n_{prism}$) is 1.486 [35]. We considered a wavelength of 2.5 μm, then the refractive index of GaS was deduced as 2.2358. The refractive index of the gold layer was calculated from the dispersion relation given by [36]:

(11) $$\varepsilon \left(\lambda \right)=\varepsilon_{r}+i\varepsilon_{i}=1-\frac{\lambda^{2}\lambda_{c}}{\lambda_{p}^{2}\left(\lambda_{c}+i\lambda \right)}$$

where $\lambda_{p}$ = 168.26 nm and $\lambda_{c}$ = 8934.2 nm.

In the case of the prism/Au/[GaS/G]^6^D[GaS/G]^6^ structure, the transfer matrix can be written as follows:

(12) $$M=\overset{N^{\prime }}{\underset{j=2}{\prod }}M_{j}=M_{Gold}{\left(M_{GaS}M_{G}\right)}^{N}M_{D}{\left(M_{GaS}M_{G}\right)}^{2N+1}=\left(\begin{array}{cc} m_{11} & m_{12}\\ m_{21} & m_{22} \end{array}\right)$$

where $N^{\prime }$ is the total number of layers forming the photonic crystal. The coefficient $\gamma_{1}$ in Equation (6) is given by $\gamma_{1}=n_{prism}\times \;cos\left(\theta_{0}\right)$ [37]. The thicknesses of the GaS, G, and defect layer were chosen as 230.9 nm, 469.1 nm, and 20 μm, respectively, and the incidence angle of light was fixed at 45°.

**Figure 13:**
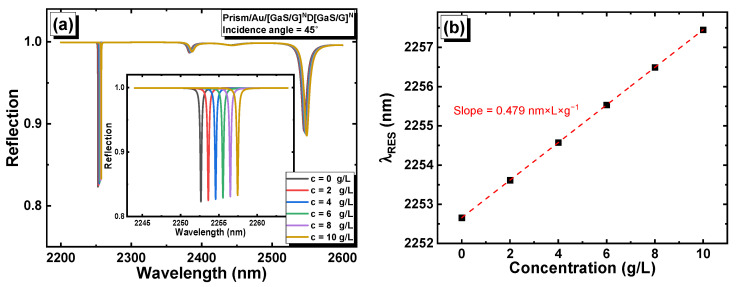
(**a**) Reflection versus the wavelength and glucose concentration in prism/Au/[GaS/G]^6^D[GaS/G]^6^; and (**b**) position of the resonance peak versus the glucose concentration.

Then, the reflection of light through the prism/Au/[GaS/G]^6^D[GaS/G]^6^ structure was calculated numerically at glucose concentrations of C = 0, 2 g/L, 4 g/L, 6 g/L, 8 g/L, and 10 g/L, as shown in Figure 13a. As can be seen in the inset of Figure 13a, the resonance peak observed at a wavelength of approximately 2252 nm shifted linearly as the concentration increased. The fitting plot of $\lambda_{RES}\left(C\right)$, shown in Figure 13b, leads to the following linear equation: $\lambda_{RES}\left(C\right)=0.479\times C+2252.66$. Lastly, the biosensing performance of the prism/Au/[GaS/G]^6^D[GaS/G]^6^ 1D PC-based biosensor was deduced and listed in Table 1. As can be seen, the biosensor exhibited a very high sensitivity of 4040 nm/RIU at the threshold value of the glucose concentration. Moreover, the FOM and Q-factor values were enhanced as compared to the structure without the prism and gold layers. The value of LOD slightly increased, as it is directly related to the working wavelength range of the biosensor. Through our calculation, we demonstrated that the biosensors reported in this study exhibit very high performance. Notably, these biosensors have broad applicability beyond glucose detection, and they can be used for other chemical and biochemical sensing applications, including blood sugar detection.

## 4. Conclusions

In conclusion, we have presented two novel designs for 1D PC-based biosensors using 2D Van der Waals materials. The [GaS/Mica]*^N^*D[GaS/Mica]*^N^* and [GaS/G]*^N^*D[GaS/G]*^N^* structures exhibited remarkable performance characteristics with respect to glucose concentration. Notably, the [GaS/G]*^N^*D[GaS/G]*^N^*-based biosensor, where G and D represent the glucose solution as target, exhibited exceptional sensitivity of 832 nm/RIU, a significant sensitivity of 832 nm/RIU, a significant figure-of-merit of 1.46 × 10^5^ RIU^−1^, a Q-factor exceeding 10^5^, and a minimum limit of detection of 3.4 × 10^−7^ RIU. Adding prism and gold layers to the [GaS/G]*^N^*D[GaS/G]*^N^*-based biosensor enhanced the sensitivity from 832 nm/RIU to 4040 nm/RIU. The successful integration of 2D materials into 1D photonic crystal structures is a promising area of biosensing research. Further research and development could lead to more advanced and more efficient biosensors for diverse applications beyond blood sugar detection.

## Figures and Tables

**Table 1:** The biosensing performance of the prism/Au/[GaS/G]^6^D[GaS/G]^6^ 1D PC-based biosensor.

Concentration	$S\;\left(\mathrm{nm}/\mathrm{RIU}\right)$	$\;\;FOM\;\times \;10^{5}\;\left({\mathrm{RIU}}^{-1}\right)$	$\mathrm{Q-Factor}\;\times \;10^{5}$	$LOD\;\left(\mathrm{RIU}\right)\;\times \;10^{-6}$
2 g/L	4040	1.80	1.01	2.77
4 g/L	4039	1.77	0.99	2.82
6 g/L	4036	1.74	0.97	2.88
8 g/L	4034	1.70	0.95	2.93
10 g/L	4032	1.67	0.94	2.99

## Data Availability

Data are available from the corresponding author upon request.
